# The Expression of Selected Factors Related to T Lymphocyte Activity in Canine Mammary Tumors

**DOI:** 10.3390/ijms21072292

**Published:** 2020-03-26

**Authors:** Joanna K. Bujak, Iwona M. Szopa, Rafał Pingwara, Olga Kruczyk, Natalia Krzemińska, Joanna Mucha, Kinga Majchrzak-Kuligowska

**Affiliations:** Department of Physiological Sciences, Institute of Veterinary Medicine, Warsaw University of Life Sciences -SGGW, Nowoursynowska 159, 02-776 Warsaw, Poland; joanna_bujak@sggw.pl (J.K.B.); iwona_szopa@sggw.pl (I.M.S.); rafal_pingwara@sggw.pl (R.P.); olakruczyk@gmail.com (O.K.); natalia.krzeminska5@gmail.com (N.K.); joanna_mucha@sggw.pl (J.M.)

**Keywords:** breast cancer model, interleukin-17, Th17 cells, co-inhibitory ligands, immune checkpoints, inflammatory cytokines, chemokine receptors, tumor-associated macrophages, cancer immunotherapy

## Abstract

Crosstalk between neoplastic and immune cells in the tumor microenvironment (TME) influences the progression of disease in human and canine cancer patients. Given that canine mammary tumors are a useful model to study breast cancer biology, we aimed to evaluate the expression of genes associated with T lymphocyte activity in benign, malignant, and metastatic canine mammary tumors. Interestingly, metastatic tumors exhibit increased expression of *CXCR3*, *CCR2*, *IL-4*, *IL-12p40,* and *IL-17*. In particular, we focused on IL-17, a key interleukin associated with the Th17 lymphocyte phenotype. Th17 cells have been shown to play a contradictory role in tumor immunity. Although *IL-17* showed a high expression in the metastatic tumors, the expression of *RORγt*, a crucial transcription factor for Th17 differentiation was barely detected. We further investigated IL-17 expression using immunohistochemistry, through which we confirmed the increased expression of this interleukin in malignant and metastatic mammary tumors. Finally, we compared the plasma levels of IL-17 in healthy and malignant mammary tumor-bearing dogs using ELISA but found no differences between the groups. Our data indicate that the IL-17 in metastatic tumors may be produced by other cell types, but not by Th17 lymphocytes. Overall, our results broaden the available knowledge on the interactions in canine mammary tumors and provide insight into the development of new therapeutic strategies, with potential benefits for human immune oncology.

## 1. Introduction

Mammary gland tumors are one of the most common cancers in dogs, especially in non-spayed females, and are still one of the leading causes of death among canines [1,2,3]. Interestingly, canine mammary tumors are an attractive and useful model for studying human breast cancer. Importantly, cancer in dogs occurs spontaneously and naturally with age. These animals share the same environment as humans and are thus exposed to similar carcinogens. Moreover, multiple clinical similarities have been demonstrated concerning the hormonal etiology of tumors, risk factors, age of onset, clinical outcomes, and prognostic factors (e.g., tumor size, metastasis presence, and clinical stage). In addition, significant analogies at the molecular level have been shown in genome-wide comparative studies of mammary cancers between human and canine. It was shown, for example, that both human and canine mammary cancers are associated with *brca1* and *tp53* gene mutations [4]. The innate and adaptive immune response of canines is also comparable to that of humans. Major subsets of the dog immune system are well characterized to have significant homology to their corresponding human subsets [5]. It is currently well recognized that a tumor mass is composed not only of transformed cancer cells but also of stroma cells (fibroblasts, mesenchymal stem cells, adipocytes, blood, and lymphatic endothelial cells) and inflammatory cells (macrophages, neutrophils, dendritic cells, T lymphocytes, and NK cells). These elements, together with signaling molecules, create the tumor microenvironment (TME) in humans as well as in dogs [6,7,8]. Continuous crosstalk between tumor and immune cells determines the progression and outcome of neoplastic disease [9,10]. Emerging evidence indicates highly conserved stromal reprogramming between canine and human mammary carcinoma, which supports the validity of using spontaneous canine mammary tumors as a model for human disease [7,11,12].

The T lymphocytes present in the TME are referred to as tumor-infiltrating lymphocytes (TILs) [13]. Three main populations of T cells have been defined, including T cytotoxic (Tc, CD8^+^), T helper (Th, CD4^+^), and T regulatory (Treg) cells. However, various subtypes can be distinguished among them, each with different functions that determine their impact on cancer prognosis and survival in dogs [14]. It was suggested that the most important negative prognostic factor is a high ratio of CD4^+^/ CD8^+^ T cells in a tumor mass both in humans and in dogs [14,15].

Tc cells, along with NK and NKT cells, can mediate the anticancer immune response since they are capable of recognizing and destroying cancer cells. Tc cells can identify the tumor-associated antigens (TAA) present in the MHC class I molecules on the surface of the target cell. Upon recognition of TAA, cytotoxic CD8^+^ T cells are able to directly kill cancer cells via perforin and granzyme secretion [16]. Elevated numbers of infiltrating cytotoxic CD8^+^ T cells is a well-established positive prognostic factor for human breast cancer as well as for mammary carcinomas in dogs [14,17,18].

Th cells, in turn, play a supportive role in the immune response through cytokine production and regulation of the recruitment and activity of effector cells for both innate and adaptive immunity. Helper CD4^+^ T cells can also recognize TAA, but they need to be displayed in the context of MHC class II molecules by professionally presenting cells, such as dendritic cells, macrophages, or B cells. Following antigen recognition, Th cells differentiate into various cell lineages, such as Th1, Th2, Th9, Th17, and Th regulatory cells, which demonstrate distinct biological functions [19]. Th1 cells exhibit expression of the T-box transcription factor (T-bet) and secrete inflammatory cytokines, including IFN-γ, TNF-α, and IL-2. These cytokines induce cytotoxic T cell expansion and increase Tc and NK cell activity against cancer. Th2 lymphocytes, on the other hand, show the expression of the GATA3 transcription factor, which regulates the production of cytokines such as IL-4, -5, -10, and -13 via Th2 cells [19,20]. Th2-mediated immunity relies on the cytokine-dependent activation of mast cells, eosinophils, B cells, and macrophages. The subsequent activity of these cells determines the role of Th2 in anticancer immunity in humans [21]. In canine tumors, higher CD4^+^ cell infiltration is related to a poor prognosis as has been observed in metastasized tumors [6,15,18].

Recently, the presence of Th17 cells in the TME of multiple human tumors was observed [22]. Th17 lymphocytes have been reported to be involved in bacterial infection and autoimmune disease pathogenesis, but studies indicate that they might also shape the immune response in human tumors [19]. Th17 cells are characterized by ROR-γt transcription factor expression and IL-17 production. However, they can also secrete IL-6, -21, -22, and TNF-α [23]. It was suggested that Th17 cells could play contradictory roles in human cancer progression [23,24,25]. On the one hand, they can mediate the antitumor immune response via the secretion of effector cytokines and the inhibition of Treg cell differentiation. On the other hand, IL-17, a signature cytokine of Th17 cells, exhibits potent proangiogenic activity, which leads to cancer metastasis [23]. In addition, Th17 cells demonstrate context-dependent plasticity, making them able to acquire the functional characteristics of immunosuppressive Treg or effector Th cells [26]. The role of Th17 cells in canine tumors has not yet been defined.

Regulatory T cells express the forkhead box P3 (FOXP3) transcription factor and secrete cytokines such as TGF-β, IL-10, and IL-35 in order to inhibit the activation of effector T lymphocytes. Treg cells also suppress immune functions through other mechanisms, including the inhibition of APC functions, the consumption of IL-2, and the production of immunosuppressive metabolites [27]. Since Treg infiltration has already been extensively studied in mammary canine tumors [28,29], we focused on the expression of other T lymphocyte-associated factors in canine mammary tumors.

Tumor-associated macrophages (TAMs) also play an important role in canine mammary cancer development [6,8]. They are different from monocytes, which are actively recruited from bone marrow by cancer cells. TAMs support tumor maintenance by enhancing the proliferation and migration of malignant cells, as well as facilitating angiogenesis extension via the secretion of growth factors, chemokines, and proangiogenic factors and switches. Moreover, it was shown that TAMs might inhibit the T lymphocyte-mediated antitumor immune response and express proteins that initiate effector immune cell death in humans and in dogs (e.g., FasL, PD-L1, and PD-L2) [30,31,32]. 

Canine mammary tumors are an attractive model for translational oncology and might be useful for the evaluation of novel diagnostic and therapeutic applications, including immunotherapy [4,7,8]. Therefore, our objective was to investigate the expression of genes encoding different molecules related to T cell activity, such as co-stimulatory and co-inhibitory ligands that regulate T cell activation, transcription factors, chemokine receptors, and inflammatory cytokines in canine mammary tumors with different malignancy statuses and metastatic potential (benign, malignant, and metastatic). We selected Th1-, Th2-, and Th17-related factors (partially based on the research by Park et al.) for canine meningoencephalitis [33]. Additionally, our aim was to characterize T lymphocyte activity-related factors, which might have importance in canine mammary tumors but are expressed not only by immune cells but also by tumor cells. Therefore, we analyzed genes whose expression is not limited to only T cells. We chose genes that encode proteins that regulate T cell activation or differentiation, such as *PD-L2*, *Gal9*, *CD86*, *CXCR3*, *CCR2*, which have not yet been investigated in canine mammary cancers. We also analyzed cytokine signatures, including *IFN-γ, IL-4*, and *IL-17* (cytokines characteristic for the Tc lineage/Th1, Th2, and Th17 cells, respectively) as well as pro- and anti-inflammatory cytokines, such as *IL-10*, *IL-12A*, and *IL-12B*. Finally, we selected *TBX21*, *GATA3,* and *RORγt* as key transcription factors for differentiation of the Th1, Th2, and Th17 cell populations, respectively. We decided to cover a wide spectrum of proteins present in bulk tumor tissue that are associated with different T cell subsets. In particular, we focused on Th17 lymphocyte biomarkers since the expression and role of Th17 cells in canine tumors has not yet been fully unraveled. We evaluated the expression of IL-17 at the mRNA and protein levels via qPCR and the immunohistochemical staining of tumor samples, respectively. We also analyzed the IL-17 concentrations in the plasma of healthy and mammary tumor-bearing dogs using ELISA. Our goal was to broaden knowledge about the interplay between malignant cells and immune cells, with emphasis on the role of Th17 lymphocytes in canine mammary cancer. We also aimed to assess potential biomarkers and therapeutic targets for canine mammary tumors which might benefit future human breast cancer clinical trials.

## 2. Results

### 2.1. Expression of Ligands Regulating T Cell Activation 

Members of the B7-CD28 family play an important role in T cell activation as costimulatory signals that simultaneously modulate the scale of the T cell response [34]. In our study, we first examined the expression of *PD-L2*, which encodes the coinhibitory ligand B7-DC (PD-L2 and CD273), and *Gal9*, which encodes galectin-9, a carbohydrate-binding protein that causes T lymphocyte dysfunction and apoptosis via its receptor TIM-3 [35,36].

**Figure 1:**
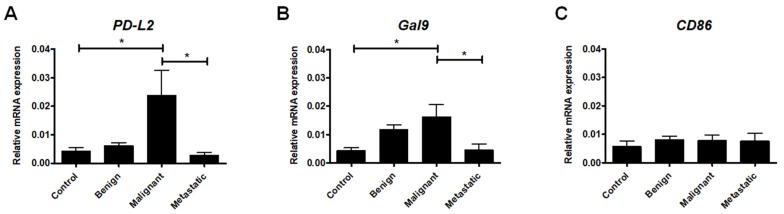
The expression of ligands regulating T cell activation at the mRNA level in canine mammary tumors. The relative *PD-L2* (**A**), *Gal9* (**B**), and *CD86* (**C**) gene expression levels in healthy mammary gland tissue (Control) and in benign, malignant, and metastatic canine mammary tumors. The results are presented as the mean ± SEM. A one-way ANOVA and Tukey HSD post hoc test were applied. Significance levels are indicated as follows: * *p* < 0.05.

We found that the expression of both *PD-L2* and *Gal9* genes was highly upregulated in the malignant mammary tumors compared to the healthy tissue. Interestingly, in the metastatic tumors, *PD-L2* and *Gal9* expression decreased significantly compared to the malignant tumors that were not metastatic. There were no significant differences between the expression levels of *PD-L2* and *Gal9* in the healthy tissue and benign and metastatic tumors (Figure 1A,B). 

We have also assessed the expression of *CD86*, which encodes the B7-2 ligand. This protein can, paradoxically, bind to both CD28 (the major co-stimulatory molecule for the activation of T cells) and CTLA4 (an important co-inhibitory receptor of T cell response) [37]. Our results show no statistically significant difference in *CD86* expression between benign and malignant or metastatic tumors (Figure 1C).

### 2.2. Expression of Cytotoxic T Lymphocyte Markers 

**Figure 2:**
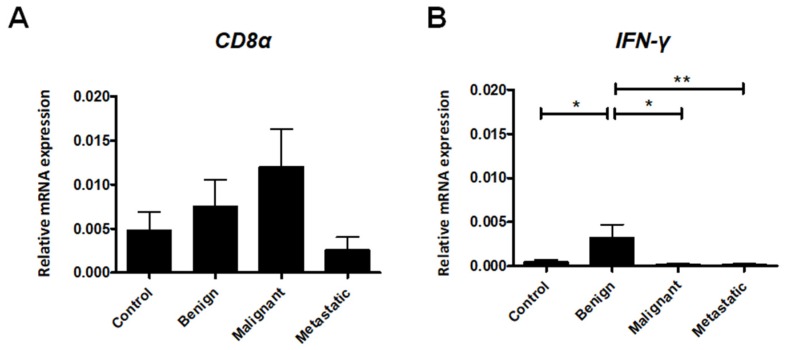
Relative gene expression of *CD8α* (**A**) and *IFN-γ* (**B**) in healthy mammary gland tissue (Control) and in benign, malignant, and metastatic canine mammary tumors. The results are presented as the mean ± SEM. A one-way ANOVA and Tukey HSD post hoc test were applied. Significance levels are indicated as follows: * *p* < 0.05; ** *p* < 0.01; *** *p* < 0.001.

Elevated numbers of infiltrating cytotoxic T cells have been well-established as a positive prognostic factor for various cancer types [17]. Therefore, we have determined the expression of *CD8α* and *IFN-γ* in canine mammary tumors. Increased expression of *IFN-γ* was observed in benign lesions compared to the malignant and metastatic tumors (Figure 2B), whereas a slight decrease in *CD8α* expression was observed in metastatic tumors, albeit without statistical significance (Figure 2A).

### 2.3. Th1 Cell-Associated Gene Expression 

**Figure 3:**
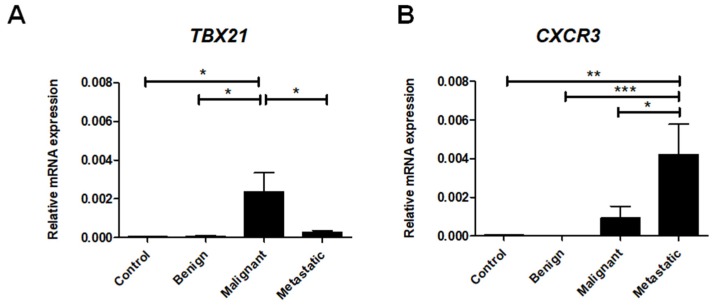
Th1 cell-associated gene expression levels in canine mammary tumors. The relative gene expression of *CXCR3* (**A**) and *TBX21* (**B**) in healthy mammary gland tissue (Control) and in benign, malignant, and metastatic canine mammary tumors. The results are presented as the mean ± SEM. A one-way ANOVA and Tukey HSD post hoc test were applied. Significance levels are indicated as follows: * *p* < 0.05; ** *p* < 0.01; *** *p* < 0.001.

In order to assess the role of Th1 cells in canine mammary tumors, we investigated the expression of *TBX21*, a gene that encodes the T-bet protein, a major transcription factor that controls the differentiation of naïve T cells into the Th1 lineage [38]. Our data demonstrate that *TBX21* expression was highly upregulated in malignant canine tumors in comparison to healthy tissue as well as benign and metastatic tumors (Figure 3A). Interestingly, we also found significantly decreased expression of *TBX21* in tumors that produce metastasis compared to the malignant non-metastatic tumors (Figure 3A). 

The CXCL9, -10, -11/CXCR3 axis regulates Th1 cell differentiation and immune cell migration to sites of inflammation [39]. Additionally, CXCR3 is expressed primarily on activated T cells, such as Th1 cells [33]. Thus, we examined *CXCR3* gene expression as it relates to the Th1 lineage. Our results show that *CXCR3* expression can be barely detected in benign tumors and healthy tissues (Figure 3B). The expression of *CXCR3* increased, however, with malignant transformation and achieved its highest expression in metastatic tumors (Figure 3B).

### 2.4. Th2 Cell-Associated Gene Expression 

**Figure 4:**
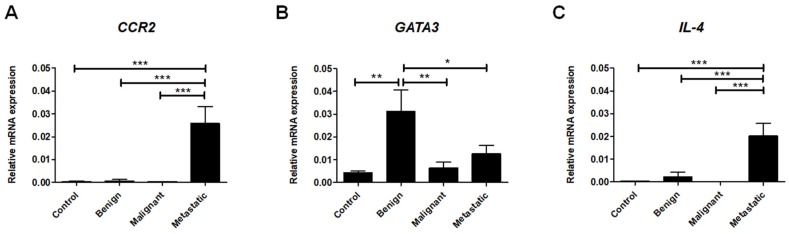
Th2 cell-associated gene expression levels in canine mammary tumors. The relative gene expression of *CCR2* (**A**), *GATA3* (**B**), and *IL-4* (**C**) in healthy mammary gland tissue (Control) and in benign, malignant, and metastatic canine mammary tumors. The results are presented as the mean ± SEM. A one-way ANOVA and Tukey HSD post hoc test were applied. Significance levels are indicated as follows: * *p* < 0.05; ** *p* < 0.01; *** *p* < 0.001.

To determine the Th2 lymphocyte phenotype in the canine mammary tumors, we assessed the expression of *CCR2*, *GATA3,* and *IL-4*. We examined the expression of *CCR2*, the receptor for MCP-1, which is involved in the development of the Th2 immune response [40]. Our data revealed the highly upregulated expression of *CCR2* in metastatic tumors compared to benign and malignant tumors as well as healthy tissue (Figure 4A). 

The expression of *GATA3*, a transcription factor responsible for promotion of the differentiation of Th2 cells and the inhibition of Th1 development [38], was found to be increased in the benign tumors in comparison to healthy tissue as well as malignant and metastatic neoplasms (Figure 4B). 

Our results also reveal that the expression of the Th2-related cytokine, *IL-4*, was highly upregulated in metastatic tumors compared to the other groups (Figure 4C). 

### 2.5. Anti-Inflammatory and Pro-Inflammatory Cytokine Expression 

**Figure 5:**
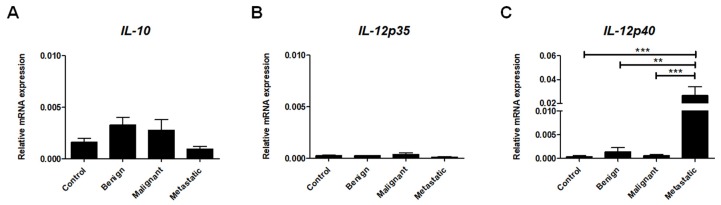
The expression of inflammatory cytokines at the mRNA level in canine mammary tumors. Relative gene expression of *IL-10* (**A**), *IL-12p35* (**B**), and *IL-12p40* (**C**) in healthy mammary gland tissue (Control) and in benign, malignant, and metastatic canine mammary tumors. The results are presented as the mean ± SEM. A one-way ANOVA and Tukey HSD post hoc test were applied. Significance levels are indicated as follows: ** *p* < 0.01; *** *p* < 0.001.

Crosstalk within the TME relies primarily on cytokine and chemokine signaling [41]. IL-10 was proven to be a potent anti-inflammatory cytokine, and is mainly produced by Treg cells but in also produced by other immune cells such as B cells, TAMs, mast cells, granulocytes, and dendritic cells as well as cancer cells themselves [42]. As shown in Figure 5A, we did not find significant differences in the expression of *IL-10* between tumors and healthy tissue or among tumors with different malignancy statuses (Figure 5A).

In contrast to IL10, IL-12, which is mainly produced by antigen-presenting cells, has been shown to exhibit pro-inflammatory effects via the induction of IFN-γ, producing Th1 and Tc cell differentiation as well as the enhancement of NK cell activity. IL-12 is a heterodimer that consists of p35 and p40 subunits. While the p35 subunit (IL-12A) is unique to the IL-12 cytokine, the p40 subunit (IL12B) is shared by both the IL-12 and IL-23 cytokines [43]. We observed marginal expression of *IL-12p35* in canine mammary tumors compared to *IL-12p40* (Figure 5B,C). We did not find significant differences in the expression of *IL-12p35* between different tumors groups and healthy tissue. By contrast, the expression of *IL12-p40* was highly increased in metastatic tumors compared to non-metastatic and benign lesions (Figure 5C). 

### 2.6. Th17cell-Associated Gene Expression

**Figure 6:**
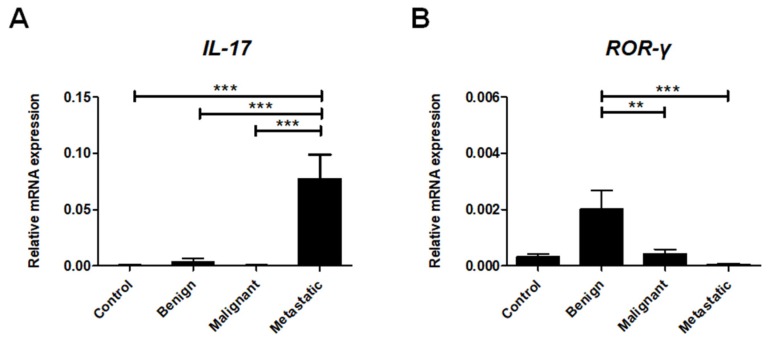
Th17 cell-associated gene expression levels in canine mammary tumors. Relative gene expression of *ROR-γt* (**A**) and *IL-17* (**B**) in healthy mammary gland tissue (Control) and in benign, malignant, and metastatic canine mammary tumors. The results are presented as the mean ± SEM. A one-way ANOVA and Tukey HSD post hoc test were applied. Significance levels are indicated as follows: * *p* < 0.05; ** *p* < 0.01; *** *p* < 0.001.

The subpopulation of Th17 lymphocytes could play dual and opposing roles in cancer progression [23]. Our study revealed very high *IL-17* expression only in the metastatic tumors. The expression of *IL-17* observed in metastatic tumors was significantly higher compared to in benign and non-metastatic malignant tumors as well as healthy tissue (Figure 6A).

Interestingly, expression of the Th17 cell-specific transcription factor *ROR-γt* was significantly increased in benign neoplastic lesions compared to malignant tumors and healthy tissue. We barely detected *ROR-γt* gene expression in metastatic canine mammary tumors (Figure 6B).

### 2.7. IL-17 Protein Level in the Canine Mammary Tumors and Tumor-Bearing Dog Plasma

Due to the significant upregulation of *IL-17* gene expression in the metastatic tumors in our transcriptomic study and given the controversial role of Th17 cells in tumor progression, we further investigated IL-17 protein expression in canine mammary tumors. 

**Figure 7:**
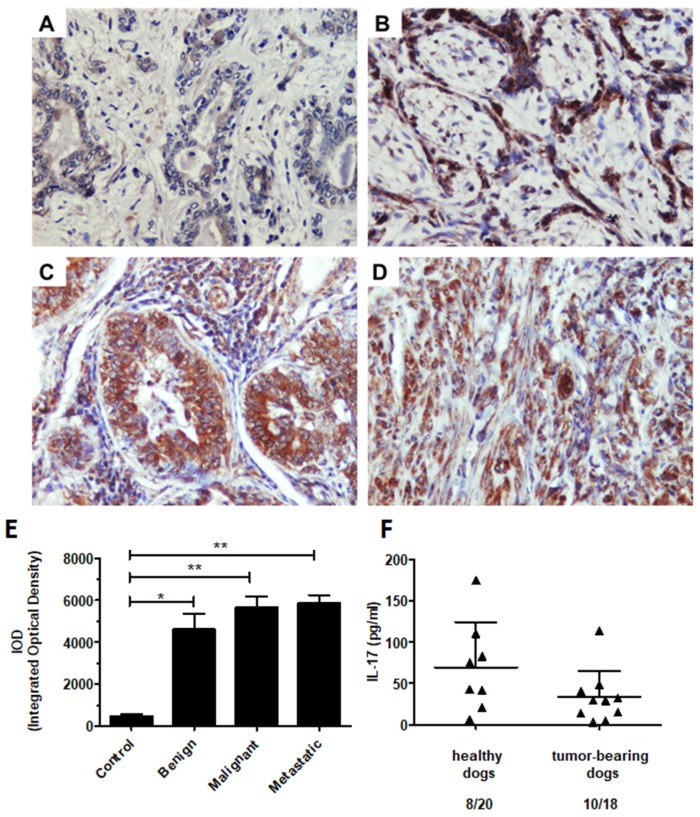
The immunohistochemical expression of IL-17 in canine mammary tumors and the plasma levels of IL-17 in healthy and mammary tumor-bearing dogs. Representative light micrographs of canine mammary gland tissue (**A**), benign (**B**), malignant (**C**), and metastatic tumors (**D**) obtained with an Olympus BX60 microscope (at 200× total magnification). The IL-17 antigen is represented by the brown-colored precipitate in the cell cytoplasm and extracellular matrix. (E) The graph showing the IOD (integrated optical density) of IL-17 expression. The results are presented as the mean ± SEM. A one-way ANOVA and Tukey HSD post hoc test were applied. Significance levels are indicated as follows: * *p* < 0.05; ** *p* < 0.01. (F) The concentration of IL-17A (pg/mL) in the plasma of the client-owned healthy (8 out of 21) and malignant canine mammary tumor-bearing dogs (10 out of 18). Student’s *t*- test was applied, and no statistical significance was observed (*p* > 0.05).

The immunohistochemical analysis demonstrated a significantly higher expression of IL-17 protein in mammary tumors compared to healthy tissue (Figure 7A–E). However, we did not find differences among the tumors at different stages of malignancy. Our data revealed comparable levels of IL-17 expression in the benign proliferative lesions and malignant tumors regardless of metastatic behavior (Figure 7E). 

In addition, we determined the concentration of IL-17 in the plasma of client-owned healthy dogs and malignant mammary tumor-bearing dogs. We did not find significant differences of plasma IL-17 level between the groups. This cytokine was detected in the plasma of 8 of 21 and 10 of 18 healthy and tumor-bearing dogs, respectively (Figure 7F).

## 3. Discussion

T lymphocytes play a crucial role in shaping the immune response against cancer. Multiple studies have demonstrated the presence of T lymphocytes in various types of mammary gland neoplasms [19]. A recent study has shown that lymphocytes comprise the primary population of inflammatory cells infiltrating mammary tumors regardless of their histological type [6]. Moreover, several studies have shown that a high number of infiltrating CD3^+^ T cells is associated with poor prognoses and lower survival rates [14,18,44]. In both human and canine mammary gland cancers, the infiltration of T cells is known to be one of the factors correlated with angiogenesis and metastasis [14,45].

Our goal was to analyze the expression of T lymphocyte-related factors, such as T cell co-stimulatory and co-inhibitory ligands, transcription factors, chemokine receptors, and inflammatory cytokines that had not yet been investigated in canine mammary tumors. 

Multiple inhibitory ligands are expressed by either cancer cells or immune cells (mainly macrophages and dendritic cells) in an attempt to suppress T lymphocyte activity [34]. We documented significantly increased expression of the co-inhibitory ligand *PD-L2* in malignant mammary neoplasms. These results suggest the action of an immunomodulatory mechanism in aggressive tumors that seeks to avoid immune surveillance. In contrast to PD-L1, which was reported to be wildly expressed in both canine immune cells and cancer cells [46], PD-L2 expression has not yet been reported in canine mammary tumors. PD-L2 is primarily expressed in dendritic cells and macrophages and, also, rarely in lymphocytes [34]. Given that malignant canine mammary tumors have been shown to be largely infiltrated by macrophages [47], we suspect that the increased expression of *PD-L2* might be linked with a high number of TAMs in canine mammary tumors. 

Interestingly, the expression of *PD-L2* and the other co-inhibitory ligand, *Gal9,* was significantly decreased in metastatic tumors compared to malignant non-metastatic tumors. Similarly, PD-L1 expression was shown to decrease in metastatic human breast cancer [48]. Both PD-L1 and -L2 are important inhibitory molecules in TME, which might have clinical significance in the prognoses of cancer patients and responses to checkpoint blockade immunotherapy [34]. In human breast cancer, PD-L2 expression was observed in 50.8% cases but was not correlated with overall survival [49]. The mechanism and relevance of altered PD-L2 expression in metastatic canine mammary tumors remains to be determined. Gal9 was demonstrated to boost antitumor immunity mediated by Th1 cells and exert anti-metastatic potential on human tumor cells through the mediation of cell aggregation [36,50,51]. Irie et al. showed that 19 out of 21 human patients with distant metastasis of their breast cancer were Gal9 negative. Moreover, the cumulative disease-free survival ratio for the patients from the Gal9-negative group was less favorable than that for the Gal9-positive patients. The reduced expression of Gal9 was also linked with the inhibition of NK cell chemotaxis and subsequent poor prognosis for colon cancer, hepatocellular carcinoma, and cervical cancer [35,51,52,53,54]. The expression of Gal9 was inversely correlated with the occurrence of distant metastasis but not with other clinical features, including local lymph node metastasis. Thus, Gal9 positive expression is proposed to be a useful prognostic factor of anti-metastatic potential in breast cancer patients [50]. Our study is the first to report a significant downregulation of *Gal9* expression in metastatic canine mammary tumors. The present results indicate that, similar to human patients, the expression of *Gal9* in dogs could potentially be used as a prognostic marker. 

We also examined the expression of transcription factors, such as *TBX21, GATA3,* and *RORγt,* which are essential for the differentiation of Th1, Th2, and Th17 cells, respectively. Our results revealed that in malignant tumors compared to benign lesions or healthy tissue, there was a much higher expression of *TBX21* encoding the Th1 cell-specific transcription factor T-bet. Interestingly, *TBX21* expression was also higher in non-metastatic malignant tumors than those that are metastatic. These odd results might be explained by research performed on a large cohort of women with breast carcinoma. This study demonstrated that although high T-bet expression is associated with adverse clinicopathologic characteristics, such as large tumor size, high histological grade, hormone receptor negativity, EGFR and p53 positivity, and a high Ki-67 index, T-bet^+/high^ tumors have a more favorable outcome and longer disease-free survival time compared to T-bet^−/low^ tumors [55]. Our results indicate that in malignant canine mammary tumors, similar to human breast cancers, *TBX21* expression might be associated with a more favorable prognosis related to a low metastatic potential for the cancer. Furthermore, these results underscore the role of infiltrating Th1 cells in antitumor immunity. 

Several studies have shown that GATA3 regulates mammary gland morphogenesis and luminal cell differentiation [56]. In addition, GATA3 expression was strongly correlated with the luminal A subtype of breast tumors, which carried the best prognostic outcome for human patients [48,49]. We found increased levels of *GATA3* in benign canine mammary tumors compared to healthy tissue as well as malignant and metastatic tumors. Our results contradict a previous study that documented the highest *GATA3* expression in canine mixed carcinoma, with the lowest level in a complex adenoma [57]. However, our results are consistent with a study performed on mice demonstrating that the loss of *GATA3* expression is associated with mammary tumors with a high grade of malignancy [58]. Likewise, in human breast tumors, low GATA3 expression was shown to be a strong predictor of poor clinical outcome, high tumor grade, positive lymph node status, and large tumor size [56]. The high expression of GATA3, in turn, was correlated with the level of estrogen receptor-α (ER), which is a highly significant marker of sensitivity to hormonal therapy in breast cancer patients [59]. Notably, GATA3 expression might be negatively regulated by T-bet. A recent study has shown that the insulin-dependent increase in the expression of T-bet in breast tumors is associated with a subsequent decrease in the expression of GATA3, followed by resistance to hormonal therapy in human ER^+^ breast cancer [60].

Data concerning ER expression in canine mammary tumors differ depending on the study. Nieto et al. showed that the immune expression of ER in dogs has prognostic value and is significantly higher in benign tumors [61]. However, Tonti et al. demonstrated that more than half of the benign and malignant canine mammary tumors negatively stained for ER, which undermines the clinical significance of ER [62]. The relationship between GATA3, T-bet, and ER expression in mammary tumors in dogs remains to be defined. 

RORγt is the principal transcription factor in Th17 cell development [23]. Since the role of Th17 cells in cancer has been specified to be a “double-edged sword” [23,25,26], we aimed to verify the expression of their major transcription factor. Our results are the first to demonstrate that *ROR*γ*t* expression is almost undetectable (very low expression in 4 out of 20 tumors) in metastatic canine mammary tumors but is upregulated in benign canine neoplasms. Interestingly, a recent study has documented that RORγ negatively regulates metastasis and aggressive tumorigenicity in human breast cancer [63]. It was shown that increased levels of RORγ are associated with better clinical outcomes. The mechanisms of RORγ activity are associated with the promotion of DNA repair and inhibition of the TGF-β-mediated epithelial-to-mesenchymal transition and mammary stem cell-related pathways. Thus, it was suggested that specific RORγ agonists may exhibit anticancer activities, targeting critical processes that drive the progression of breast cancer [63]. Further investigations of RORγt activity in canine mammary tumors are needed to assess RORγt’s utility as a therapeutic target. 

Chemokine signaling controls multiple processes during tumor progression, including tumor growth, angiogenesis, and metastatic spread [64]. We have investigated the expression of chemokine receptors, such as CXCR3 and CCR2, as associated with the development of Th1- and Th2-mediated immune response, respectively. We observed a significantly higher expression of both *CXCR3* and *CCR2* in canine mammary tumors that gave local or distant metastases. Although it was shown that the expression of CXCR3 on T lymphocytes mediates their migration to the tumor side, the presence of CXCR3 on malignant cells promoted tumor growth and dissemination. Notably, two isoforms of CXCR3 with opposite effects might be present in the TME [65]. Our results are consistent with recent meta-analyses, which have shown that higher CXCR3 expression indicates an advanced tumor stage and is correlated with the occurrence of distant metastasis in solid tumors [66,67]. Moreover, targeting CXCR3 was proposed as a strategy to prevent metastatic formation in breast cancer through the inhibition of CXCR3-dependent cancer cell migration and the enhancement of host antitumor immunity [66]. 

The CCL2/CCR2 axis in breast cancer is known to be involved in the metastatic process via the recruitment of different myeloid cell subsets, including TAMs and the induction of angiogenesis [68]. In contrast to the study that revealed no differences in CCR2 expression in the most aggressive canine inflammatory mammary cancers compared to non-inflammatory tumors [69], we documented a significant increase in *CCR2* expression in the metastatic cancers versus the benign and malignant non-metastatic tumors. Our results indicate that, similar to humans, *CXCR3* and *CCR2* expression might be associated with metastatic formation in dogs. Targeting these chemokine receptors could potentially be beneficial for canine mammary cancer patients. 

A variety of cytokines, chemokines, and growth factors are produced in the TME, thereby affecting antitumor immunity [70]. The goal of our study was to investigate whether the autocrine/paracrine cytokine network is relevant to canine mammary tumors. We determined the expression of *INF-γ, IL-4, IL-10, IL-12p35, IL-12p40,* and *IL-17*, cytokines that are related to Tc/Th1, Th2, Treg, macrophage, and Th17 cell phenotypes, respectively. The cytokine profiles of canine malignant and metastatic mammary carcinomas were described, illustrating the upregulation of IL-1, IL-6, TNF-α, and IFN-α expression [71,72]. Our study additionally revealed a significant increase in *IL-4*, *IL-12p40,* and *IL-17* expression in metastatic canine mammary tumors compared to non-metastatic tumors and healthy tissue. IL12p40 was shown to be highly expressed in breast cancer tissue. Furthermore, by binding to the IL-12 receptor, the IL-12p40 monomer can inhibit its immunological functions, thereby evading detection and reducing the eradication of cancer cells by the immune system [73]. 

Although IL-10 expression was demonstrated to be upregulated in human breast cancer as well as in inflammatory mammary carcinomas in dogs [74,75], we did not find significant differences between *IL-10* expression in healthy and neoplastic lesions, regardless of malignancy stage. 

Our study did not include the assessment of the stroma content in tumors. However, our results indicated several molecules that could serve as potential prognostic biomarkers or therapeutic targets in canine mammary tumors.

The role of Th17 cells in cancer development remains controversial [23,25,26]. We found a significantly increased expression of *IL-17* among the metastatic tumors. However, we did not detect the expression of the Th17 cell-specific transcription factor (*RORγt*) in these tumors. Thus, we anticipated the presence of other IL-17 producing cells in the canine mammary tumors. Indeed, we revealed, using immunohistochemical staining, that IL-17 is potentially produced by tumor cells themselves and infiltrates innate immune cells such as TAMs and neutrophils. 

The production of IL-17 by breast cancer-associated macrophages in humans has been previously demonstrated [76]. Further, IL-17C and IL-17E expression have been reported in human epithelial cells [77,78]. In addition, the predominance of IL-17-producing cells in breast cancer was revealed to be a poor prognostic factor linked with shorter disease-free survival in human patients [79]. A recent study reported elevated levels of Th17 cell cytokines (IL-17A and IL-17F) in T cell non-inflamed triple-negative breast cancer; thus, IL-17 was proposed as a novel prognostic biomarker for this type of cancer [80].

To evaluate the role of the IL-17 as a diagnostic biomarker in canine metastatic mammary tumors, we investigated the plasma levels of IL-17 in healthy and malignant mammary tumor-bearing dogs. We detected IL-17 in approximately half of the samples from both groups and did not find significant differences between the groups. Our results indicate that although IL-17 expression is upregulated in canine mammary tumors, this upregulation is not reflected by its concentration in the peripheral blood. On the contrary, a recent study has revealed that the plasma levels of IL-17 increase in human patients with atypical breast hyperplasia and ductal carcinoma [81].

The present data suggest that the action of IL-17 is local than systemic, and that IL-17, similarly to human studies, may be associated with the development and metastasis of canine mammary tumors. Based on the results showing increased IL-17 protein expression in canine mammary tumors compared to healthy tissue, we believe that IL-17 supports tumor growth and may exert more proangiogenic effects than the immunomodulatory activity in canine mammary tumors. Nevertheless, further investigations are needed to assess the production of IL-17 by cancer cells and the impact of IL-17 on migration and invasiveness, as well as to determine the expression of IL-17 surface receptors. 

## 4. Materials and Methods 

### 4.1. Canine Mammary Tumor Samples

Canine mammary tumor samples were collected during mastectomies from female dogs of various breeds and ages. Half of the tumor samples were fixed in 10% neutral buffered formalin and the other half of the same tumor samples were immersed in RNA*later*^®^ RNA Stabilization Solution (Invitrogen) and stored at −80 °C. For histological examination, the formalin fixed samples were dehydrated, embedded in paraffin, and processed for routine hematoxylin and eosin (HE) staining. The tumors were diagnosed according to the World Health Organization (WHO) Histological Classification of Mammary Tumors of the Dog and the Cat with further modifications [82,83,84]. The tumor samples included in our study were diagnosed as benign (simple adenomas, complex adenomas, basaloid adenoma, fibroadenoma, benign mesenchymal tumors, and benign mixed tumors, *n* = 14), malignant with no detectable metastasis (solid carcinomas, simple carcinomas, tubulopapillary carcinomas, basaloid carcinomas, and mixed carcinomas) (*n* = 18), and malignant with local or distant metastases (carcinomas, simple carcinomas, tubulopapillary carcinomas, ductal carcinomas, and mixed carcinomas), referred to as metastatic (*n* = 20). Radiography was used for the diagnosis of lung metastases. Mammary gland tissues obtained from healthy females were used as control samples (*n* = 10). In all cases, the owners’ consent was obtained for use of the canine patients’ tumor samples.

### 4.2. RNA Isolation and qPCR

qPCR was used to examine mRNA expression. RNA was isolated from tumor samples after disruption and homogenization at 50 Hz for 15 min using the TissueLyser LT (Qiagen, Hilden, Germany). RNA was isolated using a Total RNA kit (A&A Biotechnology, Gdynia, Poland) according to the manufacturer’s protocol and stored at −80 °C. The quantity of isolated RNA was measured using NanoDrop 2000 (NanoDrop Technologies, Waltham, MA, USA). The samples with adequate amounts of RNA were treated with DNaseI to eliminate DNA contamination. The samples were subsequently purified using an RNeasy MiniElute Cleanup Kit (Qiagen). Finally, the RNA samples were analyzed on a BioAnalyzer (Agilent Technologies, Santa Clara, CA, USA) to measure the final RNA quality and integrity. cDNA synthesis was performed using High-Capacity cDNA Reverse Transcription Kits (ThermoFisher Scientific, Waltham, MA, USA), according to the manufacturer’s protocol, in a Mastercycler thermal cycler (Eppendorf, Hamburg, Germany). 

The primers for *PD-L2*, *Gal9*, *CD86,* and *CD8α* were designed de novo using PRIMER3 software (free online access, http://bioinfo.ut.ee/primer3-0.4.0/) and checked using an Oligo Calculator (free online access, http://biotools.nubic.northwestern.edu/OligoCalc.html) and Primer-Blast (NCBI database, https://www.ncbi.nlm.nih.gov/tools/primer-blast/). The other primers used in our study to evaluate Th1-, Th2-, and Th17-related cytokine and chemokine receptors were reported in a a study of canine meningoencephalitis by Park et al. 2013 [33]. The sequences of primers used for qPCR are listed in Table 1. The qPCR reaction was performed using SYBR Green and a Stratagene Mx3005P QPCR System (Agilent Technologies) with an initial degeneration step at 50 °C for 2 min and 95 °C for 2 min, followed by 35 cycles at 95 °C for 15 s, an annealing step at 58 °C for 15 s, and 72 °C extension for 1 min. At the end of each cycle, the intensity of the fluorescence emitted from SYBR Green was measured. Next, the samples were subjected to a dissociation curve analysis. After the completion of this process, the samples were automatically quantified using the MxPro QPCR Software (Agilent Technologies). A single narrow peak was observed in a dissociation curve analysis at the specific melting temperature for all primers used, confirming the specificity of the observed product. Additionally, a single band of the predicted size was observed by 2% agarose gel electrophoresis for the *PD-L2*, *Gal9*, *CD86,* and *CD8α* primers, which were designed de novo by our team. *RPS19* and *HPRT* genes were used as a non-regulated reference (housekeeping) for normalization of the target gene expression. The relative mRNA expression was calculated using the comparative Ct method [85] as 2^−ΔCt^, (ΔCt = Ct_reference_ − Ct_target_). 

### 4.3. Immunohistochemistry (IHC)

Sections of 4 µm thickness were deparaffinized in xylene and rehydrated in graded ethanol. Antigen was retrieved by placing the slides in 0.02 M citrate buffer (pH 6.0) and boiling in a decloaking chamber. Subsequently, the samples were treated with a peroxidase blocking reagent (Dako, Glostrup, Denmark) for 10 min at room temperature and blocked with 5% bovine serum albumin (Sigma Aldrich, St. Louis, MO, USA) for 30 min. Next, the sections were incubated with primary rabbit antibody anti-canine IL-17 (1:200 dilution; ab79056, Abcam, Cambridge, UK) diluted in 1% bovine serum albumin overnight at 4 °C. After the PBS washing, the slides were incubated with an HRP-labeled polymer conjugated to secondary anti-rabbit antibodies from the EnVision™+ System kit (Dako). Next, 3,3-diaminobenzidine tetrahydrochloride (DAB, Dako) was used for color development. The slides were then counterstained with hematoxylin and dehydrated. Finally, the sections were mounted with mounting medium (Dako) for microscopic evaluation. A negative control test was performed by omitting the primary antibody on corresponding representative slides in each IHC experiment. Three consecutive tissue sections were examined. Around 10–20 pictures were taken of each slide (depending on the tumor size) using a BX60 microscope (Olympus, Tokyo, Japan). The colorimetric intensity of IL-17 expression, reflected as IHC-stained antigen spots (brown color), was counted by a computer-assisted image analyzer (Olympus Microimage™ Image Analysis, software version 4.0 for Windows, USA). The results are presented as the mean pixel integrated optical density (IOD) related to the color intensities of each IL-17 antigen spot.

### 4.4. ELISA

EDTA-anticoagulated whole blood was collected for diagnostic purposes from client-owned healthy dogs (*n* = 21) and mammary tumor-bearing dogs (*n* = 18) during routine veterinary procedures. All tumors were subsequently diagnosed as malignant, with adenosquamous, tubulopapillary, anaplastic, ductal, papillary, simple, complex, and mixed carcinomas. The whole blood samples were centrifuged (15 min at 2500 rpm), and the resulting supernatant (plasma) was collected and stored at −20 °C. Plasma IL*-*17 levels were measured using a Canine IL-17/IL-17A Quantikine ELISA Kit (R&D Systems, Minneapolis, MN, USA) according to the manufacturer’s recommendations. 

### 4.5. Statistical Analysis 

The statistical analysis was performed using the Prism software, version 5.0 (GraphPad Software, San Diego, CA, USA). The mRNA expression was analyzed by a one-way ANOVA followed by a Tukey honest significant difference (HSD) post-hoc test. When required (as determined by Bartlett’s test), the of data was logarithmically transformed to meet the ANOVA assumption of homogeneity of variance. The IHC data were analyzed by one-way ANOVA and Tukey HSD post hoc tests, while for the ELISA data analysis, Student’s *t*-test was applied. The data were expressed as the mean ± SEM, unless otherwise stated. Significance levels were indicated as follows: * *p* < 0.05; ** *p* < 0.01; *** *p* < 0.001.

## 5. Conclusions

Lymphocytic infiltration is an important feature of canine mammary tumors. Our data are the first to document the expression of T lymphocyte-associated factor expression, including the co-inhibitory ligands (*PD-L2, Gal9*), transcription factors (*GATA3*, *TBX21,* and *RORγt*), chemokine receptors (*CXCR3* and *CCR2*), and inflammatory cytokines (*IL-4, IL-12p40,* and *IL-17*) relevant to canine mammary tumors. However, detailed study of the stroma content of tumors would be beneficial to enrich the understanding of the specific T cell subpopulations role in canine mammary tumors biology. 

Interestingly, we revealed that the upregulation of IL-17 is not related to the presence of Th17 lymphocytes but rather to infiltration by other innate immune cells and, potentially, IL-17 production by tumor cells. Our results may have strong implications for translational oncology and the development of novel cancer immunotherapies.

## Figures and Tables

**Table 1:** Sequences of primers used for qPCR.

Gene group	Gene	Accession no.	Sequence	Product Size (bp)
Ligands regulating T cell activation	*PD-L2*	XM_847012.5	F: ACTGGCTGCTTCATGTTGTC	169
			R: CAGGTTCAAATAGCTCCGTCC	
	*Gal9*	NM_001003345.1	F: GCTGCGATTTCAAGGTGACG	134
			R: GCCTGGAGACTGGAAGCTAA	
	*CD8*	NM_001003146.2	F: GAAACCCACCCCTGATGGAG	191
			R: ACCGTACTCTTTCCTTGGTCTG	
T cytotoxic cell-associated genes	*CD8α*	NM_001002935.2	F: GTGGGTTAGACTTCGCCTGT	117
			R: CACGTCTTCTGTTCCTGTGGT	
	*IFN-γ*	AF126247.1	F: TCAAGGAAGACATGCTTGGCAAGTT	74
			R: GACCTGCAGATCGTTCACAGGAAT	
Th1 cell-associated genes	*CXCR3*	NM 001011887.1	F: TGGATGTGGCCAAGTCTGTC	200
			R: TGAGGGGGTCTCGGACCAG	
	*TBX21*	XM_548164.4	F: AAGCAGGGGCGGCGGATGTT	139
			R: ACTGCACCCACTTGCCGCTC	
Th2 cell-associated genes	*IL-4*	AF187322.1	F: TCACCAGCACCTTTGTCCACGG	96
			R: TGCACGAGTCGTTTCTCGCTGT	
	*GATA3*	XM 844060.1	F: CGAAGGCTGTCGGCAGCAAGAA	98
			R: ACGGGGTCTCCGTTGGCATT	
	*CCR2*	XM 541906.1	F: ACATGCTGTCCACATCGCA	91
			R: GGCGCGCTGTAATCATAGTC	
Anti-inflammatory and pro-inflammatory cytokines	*IL-10*	NM 0010030771	F: CAGGTGAAGAGCGCATTTAGT	107
			R: TCAAACTCACTCATGGCTTTGT	
	*IL-12A p35*	NM 001003293.1	F: TGCCTGGCCTCTGGAAAG	74
			R: TACATCTTCAAGTCCTCAT	
	*IL-12B p40*	NM 001003292.1	F: GCCAAGGTCGTGTGCCA	81
			R: CCAGTCGCTCCAGGATGAAC	
Th17 cell-associated genes	*IL-17*	NM 001165878.1	F: CACTCCTTCCGGCTAGAGAA	71
			R: CACATGGCGAACAATAGGG	
	*RORγt*	XM 540323.3	F: TCAACGCCAACCGTCCGGG	143
			R: CCGAAGCTTCCCCTTGGGCG	
Housekeeping genes	*HPRT*	NG_042858.1	F: TTATAGTCAAGGGCATATCC	104
			R: AGCTTGCTGGTGAAAAGGAC	
	*RPS19*	XM_005616513.3	F: GTTCTCATCGTAGGGAGCAAG	95
			R: CCTTCCTCAAAAAGTCTGGG	
